# Clinical practitioners’ experiences of psychological treatment for autistic children and adolescents with school attendance problems: a qualitative study

**DOI:** 10.1186/s12888-022-03861-y

**Published:** 2022-03-27

**Authors:** Johanna Melin, Markus Jansson-Fröjmark, Nora Choque Olsson

**Affiliations:** 1 Child and Adolescent Psychiatry Research Center (CAP), Stockholm County Council, Stockholm, Sweden; 2 Centre for Psychiatry Research, Department of Clinical Neuroscience (CNS), Karolinska Institutet, & Stockholm Health Care Services, Liljeholmstorget 7, 117 63 Stockholm, Sweden; 3 Department of Psychology, Stockholm University, Stockholm, Sweden

**Keywords:** Anxiety, Autism, Intervention, Psychological, Treatment, School absenteeism, School attendance problems, And school refusal

## Abstract

**Background:**

School attendance problems (SAPs) are common among children and adolescents with autism spectrum disorder (ASD). Currently, there is a lack of guidelines for treatment or interventions aimed at this group.

**Method:**

Twelve clinical practitioners were interviewed via in-depth interviews using a semi-structured question guide. Interviews were audiotaped, transcribed verbatim, and initially independently coded by two coders. The interviews were analyzed according to thematic analysis.

**Results:**

The majority of the clinicians reported that it was common that children and adolescents with ASD at CAP had prolonged SAPs. A total of four themes and 22 sub-themes were identified in the analysis: *the treatment situation; treatment goals at CAP; treatment interventions; and desired development*. Insufficient adaptations in response to core impairment of ASD and lack of support in their daily life could be factors to the development of SAPs. Prolonged social isolation in combination with severe psychiatric comorbidities was reported as a treatment barrier. Also, insufficient collaboration between mental health care services, school and social services obstructed the return to school for this group of students. Favorable factors for positive treatment outcome were: early detection, accurate assessment and coordination between mental health care and schools and environmental adaptation at school as well as at home, parent support and sometimes change of school. Concerning useful therapeutic techniques, exposure from cognitive-behavioral treatment was reported.

**Conclusion:**

Children and adolescents with ASD with limited societal support tend to develop SAPs. Important factors that impact the outcome of treatment were the length of the absence from school and the severity of psychiatric comorbidities. Tailored and adapted interventions at school, parent support and mental health care are needed. Research about assessment and treatment for children and adolescents with ASD and SAPs is needed.

**Supplementary Information:**

The online version contains supplementary material available at 10.1186/s12888-022-03861-y.

## Background

Autism spectrum disorder (ASD) is a lifelong neurodevelopmental condition. ASD is characterized by distinct differences in social communication, sensory processing, and behavioral patterns which are limited and repetitive in character [1]. About 1.6 to 1.8 out of every 100 children meet the criteria for a diagnosis of ASD [2, 3]. Co-occurring psychiatric disorders are prevalent in ASD. A recent meta-analytic review of 47 studies found that the prevalence of at least one psychiatric disorder was 54.8% [4] (e.g., the prevalence of anxiety in ASD ranged between 1.47 and 54% and depression from 2.5 to 47.1%) confirming previous results [5, 6]. There are other co-occurrent conditions, such as Attention-deficit hyperactivity disorder (ADHD), whose prevalence is higher than 30% in patients with ASD, ranging from 28 to 87% [7], and intellectual disability (ID) is prevalent approximately 35,2% in individuals with ASD [2]. Furthermore, school absenteeism may also be related to present and later decreased quality of life (QoL) [8].

According to recent studies, school children with ASD (without intellectual disabilities) appear to experience school attendance problems (SAPs) to a higher extent than neurotypical children. In Norway, 42.6% of children with ASD had SAPs in comparison with 7.1% of neurotypical children [9]. In England, higher rates of non-attendance amongst students with ASD in inclusive schools have been reported [10]. Over the years, SAPs have been described and categorized in many different ways in international research [11]. SAPs are primarily defined as overt behaviors according to a functional model, regardless of their origins [11]. It enables a description of the phenomenon on a sliding scale from complaints before going to school to completely skipping school [12]. The definition of SAPs used in this study is based on the definition presented by Maynard et al. [13]: 1) Children of school age with difficulties staying in school and with increasing absence, 2) The school absenteeism is known by the parents, 3) The parents have a wish for the child to stay in school, 4) The schoolwork is negatively impacted, and the child shows strong reluctance or worries about attendance which can be expressed through anxiety, bellyache, or some other somatic complaints, and 5) This group of children does not show signs of antisocial behavior as aggressiveness on a general basis, except possibly if they are forced to go to school.

Kearney [14] further states three criteria concerning the time aspect of absence where at least one criterion has to be met in order to be defined as SAPs [14]: a) The school student has missed 25% of the total amount of time in school during a period of two-weeks, b) The school student finds it very difficult to attend lessons during a two-week period, which causes great strain for the child, c) The school student is absent for a minimum of 10 days during a period of 15-weeks. SAPs, or any other closely linked definition, is not an independent diagnosis in the DSM-5 [1] or the ICD-10 [15].

School students with SAPs are a heterogeneous group with multiple risk factors [13], such as individual factors (separation anxiety, social phobia, specific phobia, and panic disorder with agoraphobia, depressive and emotional disorders, low self-esteem, social communication problems, chronic neurologic and somatic disorders, gaming disorder), social factors (immigration related problems, victims of adverse childhood experiences such as bullying), and educational risk factors (school difficulties) [16]. A review identified 781 risk factors for SAPs [17] indicating the need for early identification to prevent major mental health problems [18]. These factors can change over time and should not be seen as mutually exclusive. Children with ASD and SAPs are a particularly vulnerable group in need of support from the school, primary caregivers, mental health care, and others [19]. The prognosis depends on age, younger children usually are more easily treated, adolescents usually have a more prolonged course, on the other hand, recurrent problems are more frequently observed in younger children [20, 21]. In addition, school refusal, such as in relation to learning difficulties or school phobia, need to be differentiated from SAPs because it may need a different approach [22]. There is also a need for identifying co-occurrent severe anxiety problems to evaluate the priority of the intervention or treatment.

Discussions are being held concerning how schools should meet the needs of students with ASD [23]. Without specialized support during their school years, children with SAPs risk being excluded from society. Potential consequences of long-held SAPs include mental illness, addiction, social and financial-economic difficulties. Good attendance at school is closely linked with becoming an adult able to establish oneself socially, economically, and in the labor market [24–28].

There is a certain amount of research focusing on different forms of interventions concerning SAPs [13, 24, 26] such as cognitive-behavioral therapy (CBT) and psychosocial interventions to increase school attendance. A meta-analysis by Maynard et al. [13] highlighted promising results combining psychosocial interventions and CBT. Treatment focused on SAPs is described as being complicated, lengthy, and a collaboration challenge between societal organizations [29]. Contextual factors such as the family system are usually taken into account, and, therefore, multimodal treatment is common [26]. Furthermore, although several perspectives, such as those of parents [30] and school staff on SAPs in ASD have been examined [31], clinical practitioners’ perspectives have not yet been investigated.

The aim of this qualitative study was to investigate clinical practitioners’ experiences of psychological treatment for autistic children and adolescents with SAPs and psychiatric comorbidity. This study addressed several questions: (1) What are the clinical practitioners’ experiences providing psychological treatment to children with ASD and SAPs? (2) What treatment interventions or factors are considered favorable versus obstructive? (3) How do the clinical practitioners consider the treatment outcomes?

## Method

A qualitative method using face-to-face in-depth interviews was applied to assure a data-driven and inductive procedure. A semi-structured question guide was used, and data analysis was performed by using thematic analysis.

### Sample

The participants were recruited from four outpatient units at the Child and Adolescent Psychiatry center (CAP) in Stockholm. The inclusion criteria were experienced clinical practitioner working with children and adolescents with ASD with SAPS, co-occurrent psychiatric disorder and low everyday function (e.g., children who refused to visit their local care unit). A total of 12 clinical practitioners were interviewed due to saturation reached. The most common occupation was psychologists (42%), and the majority were females (67%). More than half had worked over 10 years within CAP (58%), and the majority had a CBT education (58%). Participant demographics are provided in Table 1. Inclusion criteria consisted of upholding therapeutic schooling (Systemic therapy, CBT or psychodynamic therapy); having experience providing treatment for children and adolescents with ASD, SAPs, and psychiatric comorbidity. All participants provided written consent following the Helsinki declaration. The current study received approval from the Swedish Ethical Review Authority (2018/1746–31/3).

**Table 1:** Demographic description of the clinical practitioners

Participants (*N* = 12)	Sex	Age	Basic education	Advanced training	Years of working at CAP
R1	Female	60	Nurse	CBT	10–19
R2	Male	50	Caretaker	CBT	10–19
R3	Female	66	Caretaker	CBT	10–19
R4	Female	57	Nurse	CBT, Psychosynthesis	10–19
R5	Female	35	Lic. psychologist	CBT	5–9
R6	Female	46	Behavioral science	Family therapy, Image therapist	5–9
R7	Female	55	Mental health nurse	Certified dog handler for therapy	10–19
R8	Male	55	Social worker	Family therapy	1–4
R9	Male	46	Lic. psychologist	CBT	1–4
R10	Male	36	Lic. psychologist	CBT	1–4
R11	Female	58	Lic. psychologist	PDT	10–19
R12	Female	66	Lic. psychologist	PDT, Specialist in Clinical Psychology	20–29

Note. *CAP* Child and adolescent psychiatry, *CBT* Cognitive Behavioral Therapy, *Lic* licensed, *PDT* psychodynamic therapy, social work: Bachelor of science in social work; *R* Responder

### Clinical setting

The participants were recruited from four outpatient units at CAP. Three out of 4 units had a special function of providing care in-home settings. A common group of patients were children and adolescents with a psychiatric disorder, neurodevelopmental disorder (such as ASD) with SAPs. These units provided multimodal treatment such as CBT, psychodynamic therapy, family therapy, and environmental therapy, and involved the patients’ network.

### Procedure

Clinical practitioners from four outpatient units at CAP were invited by email to participate in this study. The first author (JM) contacted the clinical practitioners who responded to the email. The practitioners were provided with information about the study and the opportunity to raise questions about participating, and JM assessed whether they were eligible to participate. Before the interview, each practitioner was asked to complete a consent form. They were informed that participation in the research was voluntary and anonymous and that each participant had the opportunity to withdraw their participation at any time. Interviews were conducted by JM between January and April 2019, as face-to-face in-depth interviews at a location chosen by the practitioners. The recording was made after taking permission from the participants at the beginning of the interviews and the records were kept confidential. The semi-structured question guide, which was based on the literature search in the area and were prepared prior to the interview with the participants. The in-depth interviews conducted using a semi-structured question guide lasted between 30 and 70 min and consisted of 10 questions from an interview guide. The first author transcribed all interviews verbatim. The semi-structured question guide starts with general questions following with specific questions (see Additional file 1). All information in the interview transcript that could be used to identify practitioners was removed (for example names, places, areas of expertise, educational backgrounds). The recordings and transcripts were kept in secure space, which only the first author could access.

### Data analysis

Thematic analysis was used to identify, analyze, and describe patterns and themes [32]. The program NVivo-11 [33] was used for the analysis. All experiences and opinions expressed by the clinical practitioners about treatment outcomes (favorable or obstructive procedures) were coded and categorized into themes. Themes that many clinical practitioners focused on were then chosen. Expressions that were more general (e.g., “It’s difficult to treat these kids”) were not put forward in favor of more specific statements (e.g., “It’s difficult to treat these kids because they play video games all the time”). Six steps for analysis were used: (1) Familiarization with the data material, (2) Generating of initial codes, (3) Search for themes, (4) Reviewing themes, (5) Definition and naming of themes, and (6) Producing the report. The first and third authors did an independent trial coding of the first interview, which was then discussed in terms of discrepancies to ensure refined coding. Then the first author continued the analysis process, while the second author reviewed it on an ongoing basis. The analysis consisted of semantic coding line by line. The codes were grouped together and categorized into sub-themes. The sub-themes were named and renamed during the process. Once coding was identified in several different sub-themes to explore representation and fit the pieces together, a thematic map was created for developing main themes that grouped the sub-themes in a meaningful way. The authors worked close to each other during the analysis to make sure that the interpretations were representative. Key quotes were ultimately chosen to represent each sub-theme.

## Results

A total of four themes and 22 sub-themes were identified in the analysis. The identified themes were: *The treatment situation*; *treatment goals at CAP, treatment interventions*; and *desired development*. For an overview of the themes containing each practitioners’ answers, see Table 2 below.

**Table 2:** Themes, sub-themes and response rate. A description of each respondents answers according to each sub-theme and the total rate of answers per sub-theme (%)

	Interview													
	1	2	3	4	5	6	7	8	9	10	11	12	Total	%
**Treatment situation**														
*SAPs and ASD*	x	x		x	x	x	x	x		x	x		9	75
*Prolonged SAPs*				x	x	x	x		x		x		6	50
*Challenging situation*	x	x	x	x	x		x	x	x	x	x		10	83
*Parental factors*	x			x	x	x	x	x	x	x	x	x	10	83
*Adjustments in school*	x	x	x	x	x	x	x	x	x	x	x	x	12	100
**Treatment goals at CAP**														
*“Our assignment”*	x		x	x	x	x	x	x		x	x	x	10	83
*Boundaries concerning treatment goals*	x	x	x	x	x	x	x		x	x	x	x	11	92
*Poor autcome*	x	x			x		x	x		x	x	x	8	67
*Frustration*	x	x	x	x	x	x	x	x			x	x	10	83
**Treatment interventions**														
*Motivational work*	x			x	x	x	x	x	x	x	x	x	10	83
*Assessment of SAPs*	x	x	x	x		x	x	x	x	x	x	x	11	92
*Behavioral activation*				x	x	x	x		x	x			6	50
*Psychoeducation*		x	x		x	x		x			x		6	50
*Exposure*		x		x	x				x	x			5	42
*Parental support*	x	x	x	x	x	x	x	x	x	x	x	x	12	100
*Collaboration*	x	x	x	x	x	x	x	x	x	x	x	x	12	100
*Change of school*		x		x	x	x	x	x	x	x	x		9	75
*ASD assessment*	x	x	x	x	x	x	x		x		x	x	10	83
**Desired development**														
*Early detection*		x	x		x	x					x		5	42
*School mapping*		x			x		x					x	4	33
*The school’ competence*		x	x	x		x					x	x	6	50
*Increased collaboration*	x	x				x		x			x	x	6	50

Notes. *ASD* autism spectrum disorder, *SAPs* school attendance problems, assessment, *SAPs* assessments of psychiatric illness and SAPS, *ASD* assessment: autism assessment, *CAP* Child and adolescent psychiatry

### The treatment situation

The clinical practitioners gave a fairly distinct picture of the treatment situation. According to the clinicians’ view, the patients’ school attendance problems were related to ASD core symptoms, school, parents or primary caregivers’ mental health problems, and a lack of collaboration between them.

### SAPs and ASD

A majority of the clinicians reported that a patient at CAP who presented extensive SAPs and psychiatric symptoms, such as anxiety or depression, had difficulties related to ASD symptoms or undiagnosed ASD.

*“The majority of my patients with SAPs have a diagnosis of ASD. It happens that I start working with a patient with for example depression or generalized anxiety disorder (GAD), but after a while when you’ve gotten to know the child and performed an assessment, then you see a lot of signs related to ASD that says yeah, we should probably do an ASD assessment.” (R4)*

### Prolonged SAPs

About half of the clinical practitioners described that their patients had been absent from school for some time, ranging between 6 and 24 months, in extreme cases up to 3 years.

*“Some have been at home for one, two years. Completely at home.” (R6)*

*“I’ve had one patient who hasn’t had any schooling for three years.” (R4)*

### Challenging treatment situation

Children and adolescents with ASD who have SAPs in combination with a long period of home isolation and psychiatric comorbidity deal with a lower level of function and symptom complexity. Almost all the clinicians described how with these circumstances, treatment was more complex and challenging. If the difficulties establishing contact were severe, this could lead to termination of treatment before a proper assessment was made. Treatment compliance differed significantly. A common scenario was meeting a teenager who had no intention of making behavioral changes. These patients often had greater difficulties imagining an alternative life. Some other risks mentioned by the clinicians were that some adolescents developed an addiction to video or computer games, which led to increased social isolation (minimized contact with others). One clinician expressed as following:

*“A child explained that he had anxiety at school and now when he was at home, that he didn’t have any anxiety, so why should he attend school?” (R5)*

### Parental factors

Parents’ mental health problems were a challenge in the treatment according to the clinicians. They described that most parents of children and adolescents with ASD and SAPs often suffered from mental health problems along with psychosocial problems. Some of them had these issues beforehand, but a majority seemed to be impaired as a consequence of the long struggle to get community support for their children with ASD.

*“The parents are often pretty exhausted after many years of uncertainty and struggle.” (R6)*

The clinical practitioners depicted that for a majority of parents involved in treatment it was difficult to implement and maintain parenting strategies.

*”...if the parents cannot maintain the learned strategies, then it becomes really difficult.” (R4)*

### Adjustments in school

All the clinical practitioners reported that there is a lack of knowledge about ASD at school and the adjustments needed in order to meet the needs of children with ASD and SAPs. According to them, adjustments made in order to accommodate the patients with ASD could entail: an increased degree of structure; clearer communication; preparation; predictability; and limitation of stimuli. The practitioners did not see that school staff have made enough efforts, such as adapting pedagogical methods to help the patient to return to the school. The schools could benefit from using for example homeschooling approaches at a slower pace, a personalized curriculum and/or connecting the children with a suitable contact person. Some clinical practitioners stated that when the school did not have an adapted approach or the framework for welcoming a child with severe SAPs and ASD back to school, the patient had nothing to gain from trying to return to the school in question. Even giving the present school a try could be considered harmful.

*“Those with really big difficulties at school, I actually almost consider it as an assault. To force them into a context that they can’t handle ( …) only results in increased anxiety and depression and self-harm.” (R4)*

### Treatment goals at CAP

Many of the clinical practitioners had opinions about how treatment goals concerning patients who did not attend school should be formulated at CAP, and how the practitioners perceived the carried-out treatments. Their answers were centered around the following sub-themes: *“our assignment”*; *“boundaries concerning treatment goals”;*” *poor outcome”* and *“frustration”*. Below we focus on the primary results.

### Our assignment

A majority of the clinicians described in various ways how they tried to be clear, both with themselves and others, that to ensure attendance at the school was not CAP’s assignment. The practitioners delineate efforts to maintain focus on what they considered to be their mission, that the child returned to a mentally stable state and a higher level of functioning. Many practitioners seemed inclined to differentiate the school’s goals from CAPs.

*“I usually say that you need a daily activity and schooling is the most natural environment for our group, but they might as well start working at the local grocery store.” (R8)*

*“It’s really good if it means that they start to attend school but (…) that’s not our original goal. That’s the schools’ goal, that the child should be in school.” (R3)*

### Boundaries concerning treatment goals

The boundaries of assignments between the schools and CAP for children and adolescents with ASD and SAPs were described as complicated. Practitioners saw school as a natural and important environment for their patients to grow and improve their mental health. A good school environment and an increased school attendance were closely intertwined with the perceived higher level of function. Almost all clinical practitioners described how they, or other practitioners in their unit, approached the task in different ways in order to get their patients back to school. Some were positive about an assignment that included school attendance, while others were more critical towards it.

*“Is this what one is educated to do, to be the person that maybe helps patients back (…) to school? Is that really what our resources at CAP should be used for?” (R6)*

*“… schooling isn’t really a part of our mission (…) but we are present at a lot of school meetings.” (R3)*

### Poor outcome

When asked how they experienced the results of treatments carried out, i.e., if the patients had reached the formulated treatment goals, most of the practitioners said that they did not reach the treatment goal if these were related to helping patients return to the school. Many practitioners described frustration when the main assignments were related to returning to school. They often found the unclear boundaries between CAP, school, and social services difficult. Many factors that affected the patients’ well-being as well as the treatment outcome were described as beyond their control.

*“Regarding a bigger presence in school? That hasn’t been successful particularly often I would say.” (R10)*

### Frustration

Many practitioners described frustration when the main treatment goals were related to school attendance. They often found the unclear boundaries between CAP, school, and social services difficult to handle. Many factors that affected the patients’ well-being as well as the treatment outcome were described as beyond their control.

*“This has been the source of massive frustration during many years.” (R2)*

*…All these cases we get concerning kids with a high absence from school is problematic because a big part of it concerns factors that’s hard for us to control. But the case is put forward to us as something for us to solve.” (R2)*

### Treatment interventions

The interventions viewed as central to progress in the treatment were motivational work, assessment of mental health and SAPs, ASD assessment, parental support, psychoeducation, cooperation with other societal organizations, behavioral activation, change of school, and exposure.

### Motivational work

Many of the clinicians described motivational work as an important intervention that had to come before initiating a process of change. During this phase, the patients had a chance to enhance their awareness about how their actions were connected to their ill-being. To make room for this phase, alongside building an alliance, was seen as crucial with children with ASD without any obvious suffering about their isolation at home.

*”…it’s pretty important to catch the small parts when (…) it’s not so fun. And try to get them to see the connection between what we do and how it effects how we feel.” (R10)*

### Assessment of SAPs

During the process to create a conceptualization with the patient almost all therapists depicted the importance of carefully assessing the patients’ school absence. To skip the phase of assessment and go straight to initiating a process of change (e.g., pushing the child back to school) was seen as hazardous. Often there was a considerable amount of stress in the system that could push the practitioners to work faster than they wished to.

*“I believe that we initiate treatment to quick before we’ve had a proper chance to assess and understand what are the underlying difficulties that we’re dealing with.” (R8)*

*“Yes, a very careful mapping of the school absence. In my experience, if one doesn’t do this it’s all a waste.” (R2)*

### Behavioral activation

About half of the practitioners depicted the importance of supporting the child and parents to break the isolation and broaden the child’s behavioral repertoire. This was seen as reasonable treatment goals in response to the common psychiatric disorders of depression and anxiety which created avoidance of many situations. This was often described as a challenging task where the therapist had to rely on the conceptualization and explore the child’s interests.

*“To help the child get out or to do other behaviors that could be useful later on when there’s a school in place.” (R10)*

*“...I actually thing that is the most important (…) this social activation.” (R4).*

*“I believe in disturbing the isolation.” (R9)*

### Psychoeducation

Many clinical practitioners mentioned providing proper psychoeducation to parents and patients as an important treatment intervention. They noticed more than occasionally a great lack of understanding of ASD, which created unreasonable demands on the child. This intervention resulted in more appropriate levels of demands on, and diminished stressors for the child.

*“…I think this is needed, that the parents get an increased understanding about autism and frankly how their children works and what they should and shouldn’t do.” (R5)*

*“There’s a lot of focus (…) to increase knowledge and the understanding about the diagnoses. It’s not the same for all children with SAPs but concerning the ASD diagnosis it’s often pretty similar I would say.” (R1)*

### Exposure

Some practitioners were hesitant to use exposure and response prevention (ERP) as an intervention of CBT. Other clinicians (trained in CBT) considered ERP useful for patients with ASD if a careful assessment of and adjustments to the functional differences was made.

*“...in my opinion, it’s harder to generalize, but it’s not impossible. So, one probably has to work at a slower pace than with an ordinary patient with anxiety. But I don’t consider autism to be another type of human where it’s hopeless and that they’ll never get anything when it comes to feelings. They are just on a continuum where they have an extra hard time with this, but everyone has emotions…So it’s not a matter of a different species.” (R9).*

### Parental support

All practitioners described working closely with the parents. Giving parental support had several layers. The patients had a low level of functioning and could be unmotivated to participate in treatment, which led to contact efforts via the adults. Parental support also led to the parents being able to give a heightened support to their child. It was described as common for the adults to feel an enormous stress about the high absence from school. This stress created relational tension. When the adults tried to make demands, it was often perceived as nagging and led to a higher level of conflict. Common interventions were to validate the parents’ stress, help them problem solve, chose their battles, and make room for conflict free moments.

*“Maybe we can help the parents to choose their battles and lessen the nagging that we know increase the level of stress.” (R3)*

### Collaboration

All practitioners described that a large part of their work was to ensure an adequate collaboration between the professionals and the parents surrounding the child. Without collaboration the patient got nowhere, in their opinion. This area was stressed by the therapist more than anything else as crucial to create a process of change. The collaboration was foremost between the parents, school, and CAP but not exclusively. In the Swedish context, collaboration with social services was common.

*“I can’t think of any patient sitting at home where we could work and make progress only by ourselves. Almost in every case there has been a collaboration with the school.” (R5)*

### Change of school

The clinical practitioners described how they could recommend a switch of schools in the event of prolonged SAPs. A change of school environment could be recommended in the following cases: substantial lack of adaptation for children and adolescents with ASD; long-term school attendance problems; the patient requires enhanced school adaptations. Most clinicians reported that according to their experience, a school change could be a powerful intervention. They stated that their patients were not able to reintegrate into their old schools, for example the child being conditioned to a negative emotion (such as anxiety) and/or to previous failures connected to the location. The clinicians found this response difficult to break.

*“It’s pretty rare to see it turn into a functioning school environment…in the same school.” (R4)*

*“When one finds another type of school with a higher degree of adjustments, that’s what I experience as most successful.” (R10)*

Often, they recommended a change to a school with more adjustments in place than an ordinary school. The practitioners spoke about many schools lack of the right knowledge to reintegrate the child again and therefore wished for a school where the staff had a greater understanding about how to adapt the school environment to a child with ASD.

### ASD assessment

In the case of an unsuccessful treatment trial (e.g., with focus on depression or anxiety) many of the clinicians emphasized the importance of making a clinical assessment to determine if the patient met the criteria for an ASD diagnosis.

*“…if there has been a long-lasting school absence for one semester and if the next semester doesn't seem to amount to any change maybe you need to question…It’s so easy to say that everything is depression and anxiety but often I think that, from my experience, that it depends on something.” (R4)*

### Desired development

The clinicians had many thoughts about desired development in this field and were eager to share how they wished to improve treatment for this group of children and adolescents. Their focus was mainly on preventing severe SAPs from evolving and the impression they had about the school’s competence.

### Early detection

Around half of clinicians considered early detection of SAPs in children and adolescents with ASD as necessary. There is a need for better competency among professionals in detecting SAPs at schools. The duration of school absenteeism was observed to correlate with the treatability of patients. The clinical practitioners, therefore, viewed the duration of SAPs as a predictor of the potential to make behavioral changes.

*“It’s not ok that so much time has passed (...). I mean, we can initiate treatments when the child has been at home for 6 months and (...) I can’t describe how cemented it is after that amount of time. And how far the way back is. It’s a disaster.” (R2)*

### School mapping

Some of the clinical practitioners expressed the need for greater routine in schools with a focus on assessment of SAPs in children and adolescents with ASD. This mapping should have a focus to assess the different factors affecting the child negatively in school, such as not knowing what to do on breaks and having to change classrooms frequently.

*“I’m of the opinion that it shouldn’t be unreasonable for the school to have a responsibility of their own for making a quick mapping. That responsibility has now landed within the psychiatric care.” (R2)*

### The schools’ competence

About half of the clinical practitioners expressed the need for greater competence and routine in schools with a focus on early detection and assessment of SAPs in children and adolescents with ASD. About 50% of the practitioners indicated that the schools they had been in contact with had a lack of knowledge about challenges that patients with ASD face at school. The clinicians expressed a need for increased competence regarding SAPs and ASD.

*”...in my opinion the schools have a poor understanding about ASD. But often they state that this is something they know well…”. (R4)*

### Increased collaboration

All therapists stated that a large part of their work was to ensure good collaboration with professionals surrounding the child. In their opinion, the cooperation between organizations around this population is crucial. About half of the clinical practitioners were not satisfied with the degree of collaboration they experienced. They wanted to see an expanded collaboration earlier in the process to have a firmer grip of the situation before the problems came to a degree that they required psychiatric care such as CAP.

*“...if we could create better cooperation, quicker, with the school, the district, social services and CAP then I think we could catch this problem so much earlier. So that it doesn’t have to become such a serious psychiatric illness that’s often the case with the children we see here.” (R6)*

## Discussion

The aim of this study was to investigate clinical practitioners’ experiences of psychological treatment for children and adolescents with ASD and SAPs. Our findings suggest that patients within the Swedish CAP with SAPs are considered, to a fairly high degree, to be suffering from a co-occurring diagnosis of ASD. These results are supported by research from the neighboring country Norway [9], which show that many school children with ASD develop SAPs. Few studies focused on children and adolescents with ASD as a group at risk for developing SAPs [13, 24, 26, 34]. King and Bernstein state in their overview that school students with learning difficulties and “communicative difficulties” are overlooked in research, which could be a way of talking about, among other groups, school students with ASD [28].

According to the definition by US authorities, as little as 15 days of absence in a school year is classified as chronic school absence [11]. Clinical practitioners describe meeting children with prolonged absenteeism sometimes lasting 2 to 3 years. Many practitioners testify that chronic school absence can lead to great difficulties when it comes to treatment in psychiatric outpatient care, which is again supported by other studies that described how chronic school absence is associated with a poor prognosis [14]. Therefore, the importance of early detection and assessment of SAPs are highlighted to prevent the absenteeism from becoming chronic.

Our results show that the clinical practitioners’ view is that there is a lack of school adaptations for children and adolescents with ASD. These findings stress the importance of 1) Adjustments in the school environments through an individualized school planning in the event of a known ASD diagnosis, and 2) The importance of early targeted interventions within the school system when symptoms of SAPs have been detected. These results are in line with the Swedish School Inspectorate (2012; 2016). The issue of early targeted interventions has been discussed in detail by Kearney [14]. There is some support in research for the view that school absence could be predicted in primary school through careful analysis and action programs [35]. The SAPs could also be predicted by identifying individual predispositions, such as separation anxiety, difficulties in making friends, and learning difficulties in autistic children to adapt the pedagogical environment and give additional support in the school.

Clinical practitioners described that a majority of schools are lacking in routines for assessing SAPs. This view is supported by Kearney et al. [14] who described that action programs for SAPs are scarce.

Furthermore, clinicians also experienced school staffs’ theoretical and practical knowledge about ASD to be insufficient, indicating the need for teaching the teachers about ASD related problems and how to handle them. This description is supported by other studies [30, 36]. A review revealed that 80% of the teachers described inadequate knowledge about ASD [14].

Regarding effective interventions, the clinical practitioners all point towards having the means and the knowledge to help these children. This could be interpreted as meaning that there is a substantial amount of ASD competence and ability to make individualized assessments and provide treatment for SAPs within CAP: possibly the same competence described as missing within the Swedish school system. The practitioners, throughout the interviews, are concerned with the boundaries between CAP and the school system. Many pointed out that on a structural level, the interventions around the school-absent child with ASD and psychiatric comorbidity are poorly organized leading to difficulties setting boundaries for treatment and resulting in poor treatment outcomes. Difficulties regarding the coordination of support between different organizations are highlighted in a Swedish investigation [37]. The importance of a high degree of collaboration and many involved adults has been described internationally [19, 29, 38, 39].

An evaluation of a Scottish project, Get It Right For Every Child (GIRFEC), with a focus on centralizing the support around the child has shown benefits regarding early intervention and effective coordination [40].

Previously, it was a relatively common perception that CBT with treatment components such as cognitive restructuring, exposure with subsequent habituation and generalizing lessons was less suitable for patients within the autism spectrum. Some voices in the conducted interviews raise doubts about working with CBT techniques. Others emphasize exposure as an effective method if adapted, taking autistic functioning into account. Cooper et al. [41] concluded that there is a need for adaptations working with ASD. A growing body of research on anxiety and ASD in children and adolescents has shown that tailored CBT is applicable and can have a good effect, such as adapted programs, e.g., BIACA [42]. On the other hand, this technique has been found to be refused by children and adolescents with ASD [43], and it needs therefore further studies to provide robust evidence. Bullying is another cause of SAPs [10].

This study has several limitations: The sample for this study was small, which limits the generalizability of the results to a larger population. Therefore, this study cannot claim to be representative for a larger group of clinical practitioners. The first author had some previous understanding of the subject, both based on theoretical training and past professional experience, which may have caused limitations in the interview context and during the process of analysis. However, this can also be a strength of the study.

The clinical implications of this paper are that it sheds light on clinical practitioners’ perspectives working with a group of patients that has not garnered much attention in research. It is a unique perspective that has not previously been investigated. This study shows the value of practitioner’s perspectives in the area of developing adequate interventions, as well as increasing the clinical practitioners’ skills and their ability to tailor the treatment, which was confirmed by a prior study [41].

## Conclusion

According to the clinical practitioners’ experiences and perspectives, children, and adolescents with ASD could be considered a group at risk of developing SAPs. Consequently, children and adolescents with ASD could benefit from early assessment within schools to identify the probability of developing SAPs. The results pointed toward a need for education among school staff, enhancing their ability to carry out assessments of SAPs as well as individualized planning for how to bring the student back to school. The schools’ competence regarding ASD was described as insufficient. Patients with ASD and SAPs often live in isolation for a long time resulting in a low level of function and widespread psychiatric comorbidity. CBT interventions, such as psychoeducation, parental support, behavioral activation, and exposure, need to be tailored and individualized for children and adolescents with ASD diagnosis as well as collaboration with professionals at CAP, school, and social services.

### Future studies

Future research should focus on designing prospective studies where school students with ASD are included. Outcome measures should entail mental health, everyday function, and school attendance. Schools should focus on implementing programs for early detection and assessment of SAPs. Studies on the impact of SAPs, preventive routines, and measures to detect SAPs in ASD at schools are needed. Further studies on collaboration between societal organizations and mental care services are needed.

## Supplementary Information

**Additional file 1.** Interview guide.

## Data Availability

The datasets generated and analyzed during the current study are not publicly available due to the lack of permission from participants to share anonymized participant data publicly but are available from the corresponding author on reasonable request.

## Abbreviations

ASD: Autism spectrum disorder
ID: Intellectual disability
ADHD: Attention-deficit hyperactivity disorder
SAPs: School attendance problems
DSM-5: Diagnostic and Statistical Manual of Mental Disorders Fifth Edition
ICD-10: International Statistical Classification of Diseases and Related Health Problems 10th Revision
CBT: Cognitive behavioral therapy
GAD: Generalized anxiety disorder
US: United States
