# Supplementary material for: Clinical practitioners’ experiences of psychological treatment for autistic children and adolescents with school attendance problems: a qualitative study
Source: BMC Psychiatry. 2022 Mar 27;22:220. doi: 10.1186/s12888-022-03861-y (PMC8958765; doi:10.1186/s12888-022-03861-y)
Supplement: Supplementary file 1 — Additional file 1. Interview guide. [file 12888_2022_3861_MOESM1_ESM.docx]

Additional file 1. Interview guide

 Your experience

1. Could you describe your competence and the number of years that you’ve work within the Child and adolescent psychiatry (CAP)?
2. Could you describe your experience of working with children and their parents where the child's problem has evolved around school attendance problems and anxiety? What does the treatment entail?
3. Is there a change in the treatment plan if the child also has an ASD diagnosis? If the treatment plan changes, please elaborate on how.

Feasible treatment interventions (children with ASD)

1. How have you work with the children’s schooling?
2. Is increased school attendance a possible treatment goal?
3. Could you describe what conditions you view as necessary to obtain an increased school attendance?
4. What treatment interventions, methods or strategies are most rewarding to bring a child with school attendance problems back to school?

Aggravating factors and treatment outcome

1. How effective do you experience CAPs treatments usually are to increase school attendance (whether an outspoken goal or not)?
2. Are there aggravating factors to the interventions you have assessed to be appropriate?
3. Do you have any wishes concerning how you would like to see this area of treatment to be developed?
4. Should CAP focus more or less on questions concerning schooling?
